# Improved quantitative accuracy in data-independent acquisition proteomics via retention time boundary imputation

**DOI:** 10.1101/2025.05.27.656394

**Published:** 2025-05-31

**Authors:** Lincoln Harris, Michael Riffle, William Stafford Noble, Michael J. MacCoss

**Affiliations:** 1 Department of Genome Sciences, University of Washington; 2 Paul G. Allen School of Computer Science and Engineering, University of Washington

## Abstract

The traditional approaches to handling missing values in DIA proteomics are to either remove high-missingness proteins or impute them with statistical procedures. Both have their disadvantages—removal can limit statistical power, while imputation can introduce spurious correlations or dilute signal. We present an alternative approach based on imputing peptide retention times (RTs) rather than quantitations. For each missing value, we impute the RT boundaries, then obtain a quantitation by integrating the chromatographic signal within the imputed boundaries. Our method yields more accurate quantitations than existing proteomics imputation methods. RT boundary imputation also identifies differentially abundant peptides from key Alzheimer’s genes that were not identified with library search alone. RT boundary imputation improves the ability to estimate radiation exposure in biological tissues. RT boundary imputation significantly increases the number of peptides with quantitations, leading to increases in statistical power. Finally, RT boundary imputation better quantifies low abundance peptides than library search alone. Our RT boundary imputation method, called Nettle, is available as a standalone tool.

## 1 Introduction

Advances in mass spectrometry (MS) sample preparation, instrumentation, and data processing have led to significant gains in the number of identifiable proteins in complex mixtures. In spite of this, we are still limited in our ability to accurately quantify proteins. One limiting factor is *missingness*, which refers to proteins that are present in the sample matrix but are not assigned quantitative values. Missingness may be the result of poor ionization, fragment ion interference, co-eluting peptides, or the inability to confidently assign peptides to spectra [1, 2]. Missingness can limit the statistical power for comparison between experimental groups or conditions. Additionally, downstream tasks such as clustering and dimensionality reduction require complete matrices of protein quantitations.

One approach to handling missingness is to remove high-missingness proteins from downstream analysis. Thresholds for determining whether to exclude a protein are arbitrary, but range from 50% missingness, often used in proteomics [3, 4], to 80%, common in metabolomics [5, 6]. This practice can remove signal from potentially interesting proteins, especially low-abundance proteins, which are more likely to be missing [7]. Another approach to handling missingness is “plug-in” imputation, in which statistical or machine learning methods are used to estimate missing values based on the observed values alone. Imputed quantitations are plugged directly into the matrix of observed peptide or protein quantitations. Popular plug-in methods include low value impute [8], *k*-nearest neighbors (kNN) [9] and random forest [10, 11]. Despite its ubiquitousness, plug-in imputation can introduce spurious correlations between proteins and samples [11, 12], as well as noise, diluting the biological signal.

We address missingness in data-independent acquisition (DIA) proteomics by imputing retention times (RTs) rather than peptide or protein quantitations. We start by generating a matrix of RT boundaries for all peptides across all MS runs in an experiment. We impute missing RT boundaries and write the imputed RTs to a spectral library bibliospec, or blib, file. We then import the blib and corresponding raw files into Skyline [13] and perform peptide-level quantitation. For previously missing peptides, the extracted ion current (XIC) within the imputed RT window is integrated to obtain a nonzero signal. The key advantage of our approach is that missing values are replaced with real measured quantities, rather than estimates based on other measurements.

To demonstrate the utility of our RT boundary impute approach—called Nettle—we apply it to three DIA datasets. These are: (i) a dataset obtained from Alzheimer’s disease (AD) clinical samples, derived from the superior and middle temporal gyri (SMTG) brain regions [14], (ii) a matrix matched calibration curve (MMCC) dataset for a bead-based serum assay [15], and (iii) a biodosimetry dataset obtained from mice exposed to varying doses of ionizing radiation. We first show that Nettle increases the number of differentially abundant (DA) peptides identified between autosomal dominant disease (ADD) and cognitively normal control AD samples. These include peptides from canonical and emerging AD markers such as APP, APOE, MDK and COL25A1. We focus exclusively on peptide quantitation, as protein roll-up can hide missingness at the expense of data richness. AD is a disease of aberrant proteoforms, and rolling up to the protein level can mask differential signal from important proteoforms such as amyloid precursor protein (sAPP-*α* and sAPP-*β*) [16], which eventually give rise to amyloid plaques [17].

We then benchmark Nettle against popular plug-in impute methods and show that Nettle produces the most accurate peptide quantitations. We also show that Nettle improves biodosimetry, or the ability to estimate ionizing radiation exposure in biological tissues [18]. Finally, we use the MMCC data to show that Nettle reduces peptide lower limit of quantification (LLOQ) relative to library search alone. Nettle is an open-source implementation of RT boundary imputation and is available on GitHub (https://github.com/Noble-Lab/nettle).

## 2 Related Works

Traditional plug-in imputation is performed on “quant matrices” in which one dimension corresponds to fragments, peptides, or proteins and the other dimension corresponds to MS runs or demultiplexed TMT channels. These methods typically apply statistical or machine learning (ML) approaches to estimate missing values based solely on the observed values in the quants matrix. Such methods can be thought of as “plug-in” in the sense that imputed values are directly plugged into the quants matrix and used in downstream analysis alongside the observed values. The canonical example of plug-in imputation is the Perseus strategy of low value imputation [8], in which missing values are replaced by random draws from a Gaussian distribution centered on the lowest observed value in that MS run. MissForest, an approach based on fitting a separate random forest regression model to each missing value [10], is one of the most accurate plug-in imputation approaches [11]. Recently, deep learning (DL) models such as Lupine [19] and PIMMS [20] have emerged, which use deep neural networks (DNNs) to learn patterns of missingness and perform plug-in imputation. While powerful, DL-based methods have longer run times and require specialized hardware (i.e., graphical processing units or GPUs) and technological know-how not available to most labs.

## 3 Methods

### 3.1 Data acquisition and processing

We obtained mzML files from two previously published studies: Merrihew et al. 2023 [14] and Wu et al. 2024 [15]. The first was a study of individuals enrolled in the Adult Changes in Thought (ACT) and the University of Washington (UW) AD Research Center (ADRC). Individuals were assigned to four categories based on behavioral, genetic and pathological evidence. “Autosomal dominant disease” (ADD) individuals had causal mutations in either PSEN1, PSEN2 or APP. “Sporadic” individuals exhibited AD-associated neuropathologic change but did not have causal mutations. “Control” individuals exhibited neither AD-associated neuropathologic change nor AD-associated behavioral symptoms. For our purposes, we focused on samples obtained from superior and middle temporal gyri, or SMTG, brain regions.

Wu et al. 2024 developed a bead-based protocol for enriching and capturing membrane-bound particles in plasma. The enrichment strategy, called Mag-Net, was followed by DIA proteomics and was able to differentiate between AD and non-AD plasma proteomes with high accuracy. For our purposes, we focused on an MMCC assay developed for Mag-Net. mzML files for AD and MagNet were obtained from Panorama [21].

We also obtained mzML files from an unpublished biodosimetry study. These data were part of the Intelligence Advanced Research Projects Activity (IARPA) Targeted Evaluation of Ionizing Radiation Exposure (TEI-REX) challenge. The goal of this challenge was to develop an assay capable of predicting the dose of ionizing radiation received by biological tissues (notice ID: W911NF22S0002).

mzML files were searched with DIA-NN v1.8.2 [22] using the following settings: *unimod4*; *qvalue* 0.01; cut K*, R*, !*P; *reanalyze*; *smart-profiling*. Spectral libraries (.blib), generated with DIA-NN, were converted to CSVs in which rows correspond to transitions and columns correspond to RT start or RT end for each MS run.

### 3.2 RT boundary i mputation

We developed Nettle, a method for RT boundary imputation. The starting point for Nettle is the previously described RTs matrix. As a preliminary processing step, the highest and lowest 1% of RTs were replaced with NaN (not a number), under the assumption that they primarily correspond to contaminants. Missing values in the trimmed RTs matrix were then imputed with distance-weighted kNN, with *k* set to 8. The scikit-learn (v1.5.2) KNNImputer implementation was used. The distance metric was Euclidean; peptides were features and runs were samples. The imputed RTs matrix was then written back to a blib. The imputed blib was read into a Skyline document along with the original mzML files and the FASTA used during library search.

For the AD and TEI-REX datasets, the following Skyline settings were used: *Normalization Method:* Equalize Medians; *MS level:* 2. For the MMCC dataset, the following Skyline settings were used: *Regression Fit:* Bilinear; *Normalization Method:* None; *Regression Weighting:* None; *MS level:* 2; *Calculated LOD by:* Bilinear turning point. We exported Skyline reports of peptide abundances, where abundance refers to the sum of peak areas for all transitions of a given peptide, after background subtraction. RT alignment was not performed. We reasoned that a clustering algorithm such as distance-weighted kNN should be able to account for systematic differences in RTs between samples without explicit RT alignment. The Skyline documents used in this study can be found in Panorama public (https://panoramaweb.org/GS4ghw.url).

## 4 Results

### 4.1 RT boundary imputation enables quantitation of additional AD peptides

It can be challenging to accurately identify peptides or proteins that differ quantitatively between experimental groups, especially when the number of samples in each group is relatively small. Missingness is part of the reason: if a peptide is completely missing in one group, then it is impossible to calculate a quantitative ratio for it. We hypothesized that RT boundary imputation could address this problem. To test this hypothesis, we identified DA peptides between ADD (*n* = 24) and cognitively normal control (*n* = 9) samples, with and without RT boundary imputation. In each case, we performed two-sample t-tests followed by the Benjamani-Hochberg (BH) correction. DA peptides were defined as *log*_2_ fold change >1.0 and BH-corrected p-value <0.01.

**Figure 1.**
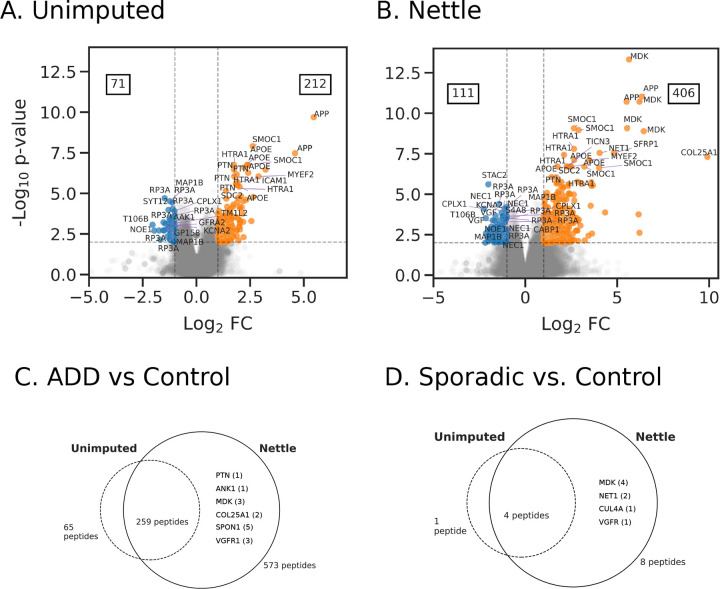
RT boundary impute identifies additional differentially abundant peptides in Alzheimer’s disease. Peptides have been annotated with their gene IDs. **A)** ADD (n=24) vs. control (n=9) samples, library search alone. **B)** ADD vs. control samples, library search + Nettle imputation. Benjamin-Hochberg corrected p-values are reported. The genes associated with the top 25 DA peptides are annotated. **C)** ADD vs. control samples, venn diagrams of up-regulated peptides identified with library search alone vs. library search + Nettle. **D)** Sporadic vs. control.

Figure 1A–B shows that more quantitatively different peptides were identified after RT boundary imputation (406 up-regulated, 111 down-regulated for Nettle; 251 up-regulated, 89 down-regulated for unimputed). These peptides correspond to canonical AD genes such as APOE, TAU and APP [17, 23], as well as emerging markers such as SMOC1 [24] and MDK [25].

The genes associated with the top 25 DA peptides are annotated in Figure 1A–B. We then examined the overlap between unimputed and Nettle imputed DA peptides, for sporadic vs. control and ADD vs. sporadic (Figure 1C–D). In both cases, Nettle identifies a larger set containing the majority of the unimputed DA peptides. But Nettle detects additional peptides mapping to AD associated genes such as MDK/PTN [25], ANK1 [26] and SPON1 [27, 28]. Additionally, the total number of quantified peptides in each experimental group increased after RT boundary imputation (Supplementary Figure 2B).

**Figure 2.**
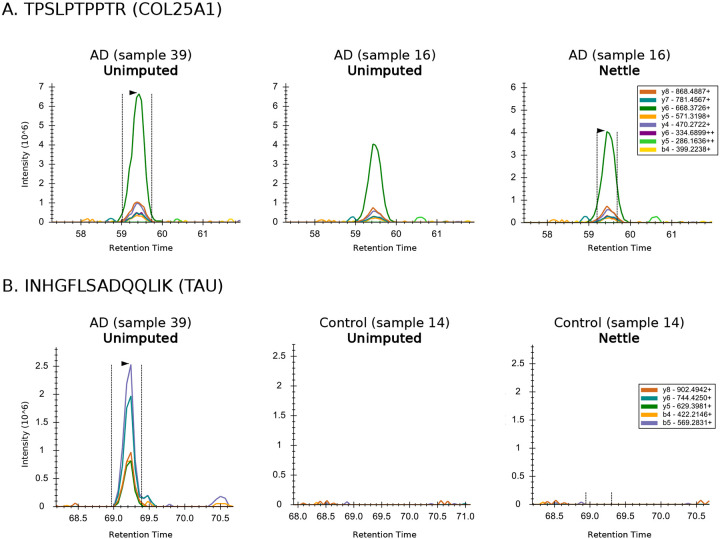
RT boundary impute enables quantitation of Alzheimer’s disease peptides. **A)** Chromatographic peaks for a peptide of COL25A1 (Collagen-Like Alzheimer Amyloid Plaque). Left: an ADD sample. Right: a control sample. **B)** Same as A, after Nettle. **C)** Chromatographic peaks for a peptide of TAU. **D)** Same as C, after Nettle imputation. Vertical dashed lines and arrows indicate whether a quantitation was obtained for that peak.

To further validate Nettle’s results on this dataset, we examined chromatograms for two tryptic peptides, INHGFLSADQQLIK and TPSLPTPPTR, of COL25A1 (Collagen-Like Alzheimer Amyloid Plaque) and TAU, respectively. COL25A1 is a brain-specific membrane collagen that is implicated in Amyloid-*β* plaque formation [29]. TAU is a microtubule protein that undergoes complex post-translational modification and splicing and can manifest as aberrant neurofibril tangles [17]. INHGFLSADQQLIK is only DA after RT boundary impute with Nettle. As shown in Figure 2A, this peak is clearly present in ADD samples (left) but absent in control samples (right). DIA-NN does not produce a quantitation for the control samples; this is a missing value. However, Nettle borrows information from other MS runs to draw reasonable RT boundaries and integrate background signal for the control samples (Figure 2B), assigning a (very low) quantitation to this peptide. Figure 2C shows that DIA-NN misses a well-defined peak for the peptide TPSLPTPPTR. Nettle draws reasonable peak boundaries and produces a quantitation for this TAU peptide, which could be important for AD progression.

### 4.2 RT boundary imputation improves quantitative accuracy

Having established that RT boundary impute increases the number of quantified peptides, we next sought to address the accuracy of those quantitations. MMCC data were used for this analysis. An MMCC experiment is a serial dilution series in which human sample is diluted with increasing concentrations of non-human background (in this case chicken) [30]. The background is “matched” in terms of matrix complexity, to prevent undesirable matrix effects during LC and electrospray. The advantage of this approach is that the ratios of peptide abundances between samples are known and can serve as ground truth. The expected quantitative ratio for a peptide A is obtained by dividing its intensity in a given sample by its intensity in the 100% (i.e., undiluted) sample: *log*_2_(*A/A*_100_). We imputed missing RT boundaries with Nettle, obtained quants reports from Skyline and assessed how close the *log* ratios are to the expectation.

Using the MMCC dataset, we compared the quantitative accuracy of RT boundary to plug-in imputation. We selected three plug-in imputation methods: low value [8], kNN [9] and MissForest [10]. “Low value” replaces missing values with the lowest observed quantity for that peptide in any sample across the experiment. kNN is one of the most commonly used plug-in methods [11]; it consists of identifying the *k* most similar MS runs, averaging them and imputing missing values with the mean quantities for each peptide. MissForest is one of the best performing plug-in imputation methods [11]. For every missing value, MissForest fits a random forest regression model to the observed values, makes a prediction and proceeds to the next missing value. The predicted value is then treated as observed and is included in the training set for the remaining regression models.

**Figure 3.**
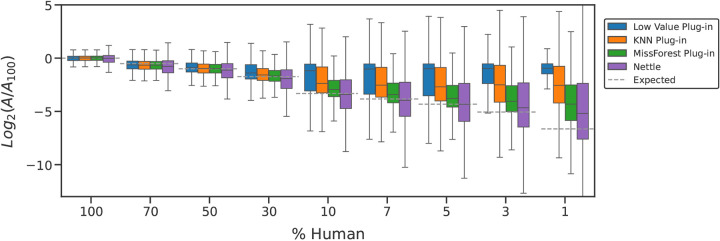
RT boundary imputation produces the most accurate peptide quantitations. For an MMCC experiment. Missing values were handled with either plug-in imputation (low value, KNN, MissForest) or RT boundary imputation with Nettle. Peptide abundances were normalized to the abundance in the 100% (undiluted) samples (i.e., *A*_100_); *log*2 ratios are reported. The horizontal dashed lines indicate the expected ratios for each sample.

Figure 3 suggests that Nettle produces more accurate peptide quantitations than the three plug-in methods. That is, the peptide quantitations are closest to the expected *log* ratios after RT boundary impute with Nettle. It is important to note that an MMCC experiment is designed to stretch sample prep methods, instruments and analytical tools to their breaking point. It is unreasonable to expect any experimental workflow to perfectly capture the expected log ratios, especially for the extreme dilution conditions (e.g., 1%, <1%). At such extreme dilutions, it becomes very difficult to differentiate chromatographic signal from background noise. Thus, the important takeaway is that Nettle comes closest to the expected *log* ratios.

Additionally, RT boundary impute significantly increases the number of peptide quantitations (Supplementary Figure 2A). In the 1% dilution, 23,339 peptides are quantified with Nettle, compared to only 5,193 without. This corresponds to a 77% increase in quantitative peptides. While many of these quantities are just integrated background signal, these peptides now have quantitations—corresponding to actual measurements—and can be included in DA analysis. Without quantitations, these peptides would be missing and would be either excluded from analysis or imputed with plug-in methods.

**Figure 4.**
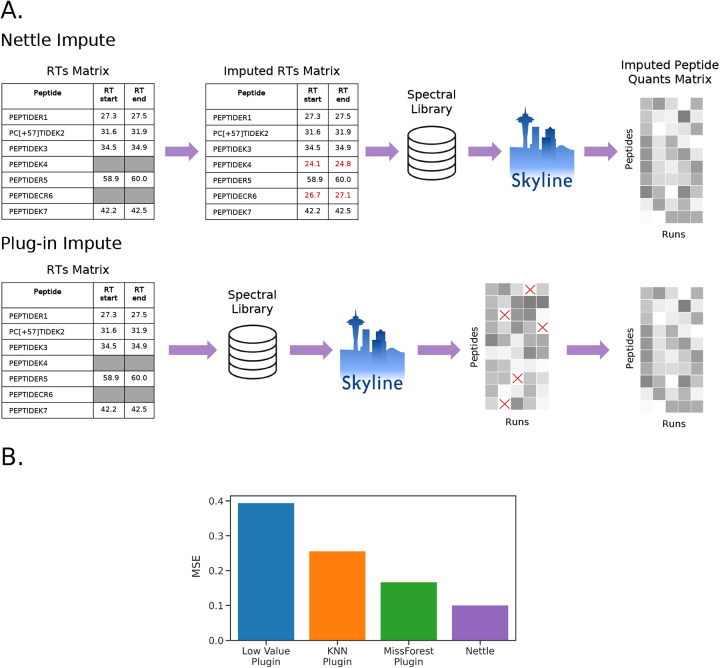
Comparing quantitative accuracy of plug-in to RT boundary imputation with a hold-out experiment. For the Alzheiemer’s disease dataset. **A)** Schematic. The same transitions are masked in the spectral library and imputed with either Nettle or plug-in methods. **B)** The test set reconstruction accuracy for each method.

One potential limitation of MMCC is that it doesn’t resemble “real” MS proteomics data. That is, we should not expect to see precise ratiometric relationships between samples derived from biological tissues. To address this limitation, we performed a hold-out experiment on the AD dataset. In the spectral library-derived matrix of RT boundaries, 20% of start-end pairs were randomly masked (Figure 4A). One version of the RTs matrix was imputed with Nettle, and the other was left unimputed. Skyline was then used to obtain peptide-level quantitative reports. In the plug-in case, this report was then imputed with either low value, kNN or MissForest. The mean squared error (MSE) between observed and imputed quantitations was then compared (Figure 4B). Lower MSE indicates more accurate estimates of the held-out peptide quantitations. Because the same set of peptides was held out in both cases, the results are directly comparable. The results (Figure 4B) again indicate that Nettle produces more accurate quantitations than plug-in methods.

**Figure 5.**
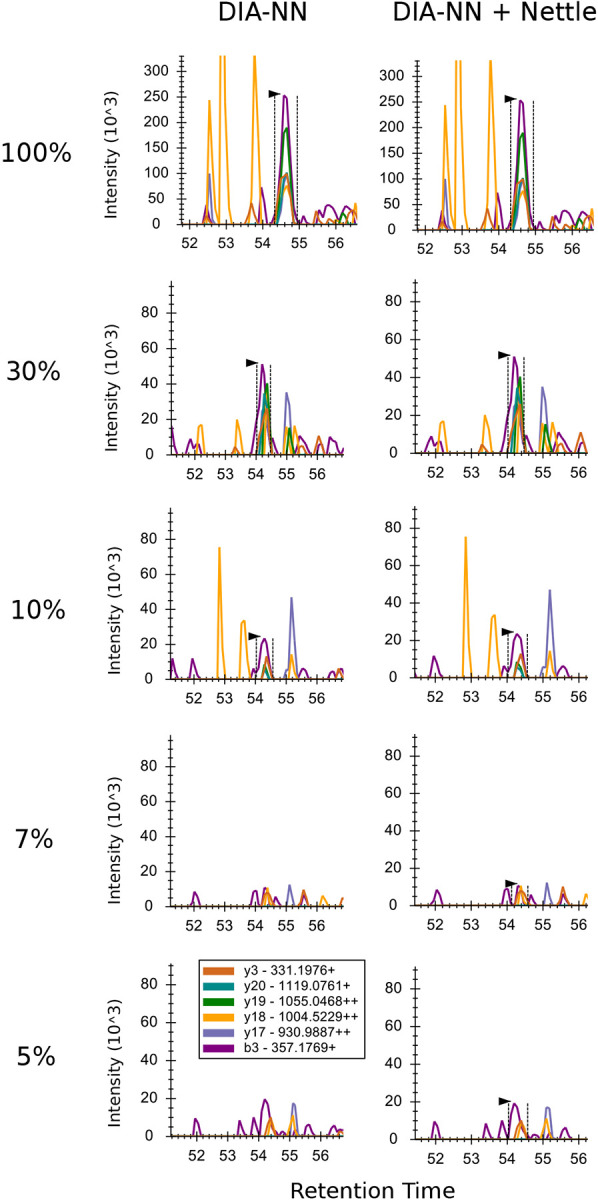
Chromatograms for the peptide DQLQTFSEEHP at different dilutions in the MMCC experiment. In both columns, library search was performed with DIA-NN. On the right, missing RT boundaries were imputed with Nettle. Percentages on the left indicate the fraction of human peptides in the sample. Vertical dashed lines and arrows indicate whether a quantitation was obtained for that peak.

We manually inspected the Nettle imputed RT boundaries for the MMCC dataset. We started with the 100% (undiluted) condition, in which the chromatographic peaks should be very well defined, then moved to lower and lower dilutions. Figure 5 shows this result for the tryptic peptide DQLQTFSEEHP. In the undiluted sample, the peak is well defined and identified by both DIA-NN (left) and DIA-NN + Nettle (right). This is also true in the 30% and 10% conditions. But in the 7% condition, DIA-NN fails to identify the peak, even though it is clearly present, whereas Nettle draws reasonable boundaries around this putative peak. This is also true for the 5% and 1% conditions. While Nettle may identify background signal in the extreme dilution samples, it still assigns low intensity quantitative measurements to those peptides.

Finally, we assessed the distributions of RT boundary imputed quantitations. Missingness in MS proteomics is a combination of missing not at random (MNAR) and missing completely at random (MCAR), with MNAR typically accounting for the majority [7]. Lower abundance peptides are more likely to be missing. We hypothesized that imputed peptide quantitations should be left-skewed relative to the observed. This is indeed the case for all three datasets (Supplementary Figure 3).

### 4.3 RT boundary imputation improves biodosimetry

We reasoned that RT boundary imputation might improve the performance of classification or regression models that distinguish between groups of samples. Fitting such models is common practice in proteomics, for example, classifying tumor samples by phenotype to identify potential drug targets [31], identifying coexpression modules in AD samples [32], and associating protein biomarkers with sex in lung disease [33]. The particular use case we chose was biodosimetry, or the ability to estimate ionizing radiation exposure in biological tissues [18]. We obtained data from the IARPA TEI-REX challenge to test our hypothesis. We again compared Nettle to plug-in imputation.

The goal of the IARPA TEI-REX challenge was to design an assay for predicting radiation exposure dosage from non-invasive samples (e.g., skin disks), particularly for low dosages (e.g., <1 Gy). The challenge consisted of two parts: in the first, we obtained high exposure (1–4 Gy) mouse samples and conducted a DIA proteomic workflow. We then trained an ensemble of gradient boosted decision trees to predict radiation dosage. This analysis allowed us to select a target panel of 92 proteins, corresponding to 1,218 peptides, with high predictive power. In the second part of the challenge, we conducted the same DIA proteomic workflow on a set of low-exposure (<1 Gy) samples. We then fit an elastic net regularized regression model using the 1,218 peptides in our target panel to predict radiation dosage. The median intensity of the target peptides was *e*^10.9^, compared to the median of all quantified peptides *e*^12.0^. The missingness among target peptides was 19%, compared to 12% among all peptides.

**Figure 6.**
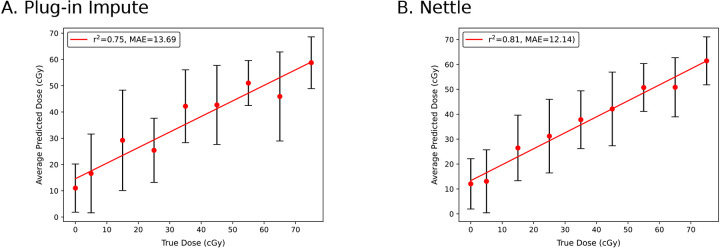
RT boundary impute improves predictions of radiation dosage from DIA proteomic profiles. Elastic net regularized regression was fit to a panel of 92 informative peptides. Regression *r*^2^ and median average error (MAE) are indicated. Best fit lines are shown in red. Error bars indicate one standard deviation from the mean predicted dose. **A)** Missing values were imputed directly from the peptide quantitations with kNN. **B)** RT boundary imputation was used to address missingness.

Considering the relatively low intensity and high missingness among target peptides, we reasoned that this analysis would benefit from imputation. We performed plug-in imputation with kNN (*k*=3) and RT boundary imputation with Nettle. 10-fold cross validation was performed. The result is shown in Figure 6: the median average error (MAE) between the predicted and true radiation dose decreased from 13.69 for plug-in to 12.14 for Nettle. The correlation coefficient increased from 0.75 to 0.81. We speculate that Nettle improved our ability to quantify low abundance target peptides, leading to improvements in biodosimetry prediction.

### 4.4 RT boundary imputation quantifies low abundance peptides

Many biologically interesting proteins are low abundance, including neurotransmitters in brain, tumor biomarkers in blood, and signaling and transcription factors in all tissues [15]. However, low abundance peptides are more likely to be missing [7], which can make it challenging to obtain quantitative ratios between groups of samples for these peptides.

We used the MMCC dataset to assess the quantitative accuracy of low abundance peptides. In this context, peptide LLOQ is defined as the lowest analyte concentration at which linear increases in signal (i.e., MS2 peak areas) correspond to proportional increases in analyte concentration [30]. It is the lowest concentration at which we can confidently quantify a peptide. This is distinct from the limit of detection (LOD), which is the concentration of a peptide required for identification. LOD is typically less than LLOQ. To calculate peptide LLOQ for a calibration curve, Skyline fits a piecewise regression model to the noise segment (the intensity range for which there is not an increase in signal proportional to increase in analyte abundance) and the linear segment, identifies the LOD based on the standard deviation of the noise segment, then bootstraps the linear segment and looks for the quantity at which CV within the resampled data is <20% [30].

**Figure 7.**
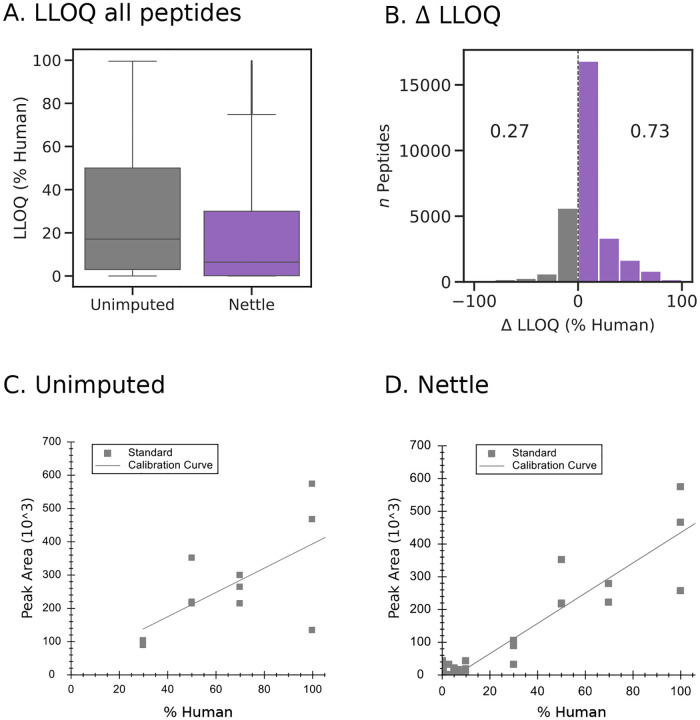
RT boundary imputation reduces peptide LLOQ. For the MMCC experiment. **A)** LLOQs for all peptides, for library search alone vs. library search + Nettle. The % human (relative to non-human background) at which the peptide becomes quantitative is indicated. **B)** Change in LLOQ (unimputed LLOQ − Nettle imputed LLOQ) for all peptides. **C)** Calibration curves for the peptide TQTHATLC[+57]STSAK without imputation, and **D)** after Nettle imputation. Calibration curves were generated in Skyline.

RT boundary imputation reduces peptide LLOQ. The median LLOQ for all peptides with library search alone is 17.1%, compared to 6.4% after Nettle (Figure 7A). Here, LLOQs are expressed as the percentage of human sample relative to background at which a given peptide becomes quantitative. Additionally, Nettle reduces the LLOQ for 73% of peptides (Figure 7B), with the largest fraction falling in the 0–20% reduction bin. We used Skyline to plot calibration curves for TQTHATLC[+57]STSAK, a peptide with a representative reduction in LLOQ with Nettle. With library search alone, this peptide becomes quantitative when the percentage of human sample relative to background is 21.2; with Nettle, it becomes quantitative at 13.8% (Figure 7C–D). Nettle is able to accurately quantify samples below the 20%:80% dilution, leading to a significantly lower LLOQ. Overall, this result indicates an increased ability to quantify peptides at low concentrations.

## 5 Discussion

Missing values limit our statistical power to draw conclusions from quantitative protemic datasets. Missing values are traditionally handled by plug-in imputation, in which statistical or machine learning models are used to estimate missing values from the observed values alone. Our retention time boundary imputation approach differs in that missing values are filled in with measured quantities—the extracted ion current within an imputed RT window—rather than estimated ones. We hope that this feature, along with the accuracy and capabilities demonstrated here, will encourage the adoption of RT boundary imputation for DIA proteomics.

RT boundary imputation with Nettle improves quantitative accuracy. We demonstrate this on an MMCC dataset with known ratiometric relationships between samples and find that Nettle produces peptide quantities that are closer to the expectation than plug-in methods (Figure 3). This is further demonstrated with a hold-out experiment on a dataset composed of AD patient samples, in which Nettle produces the lowest reconstruction error (Figure 4). Equally important, Nettle enables quantitation of additional DA peptides between ADD and control samples, from genes with known AD association including APOE, TAU, APP, COL25A1, SMOC1 and MDK (Figure 1). Chromatographic evidence (Figure 2) reveals that library search alone is unable to quantify the peptides INHGFLSADQQLIK and TPSLPTPPTR—from COL25A1 and TAU—in a number of samples. Nettle assigns quantitations to INHGFLSADQQLIK and TPSLPTPPTR, allowing us to derive quantitative ratios between ADD and control samples for these peptides. Nettle increases the number of quantified peptides in an MS run (Supplementary Figure 2), corresponding to gains in statistical power and alleviating the need to throw out low abundance, high-missingness peptides. Finally, we demonstrate that Nettle improves the ability to predict the dosage of ionizing radiation in mouse pelt samples.

Nettle is an open-source, standalone software package meant to function alongside the Skyline data analysis platform. The improved quantitative accuracy offered by Nettle, alongside a highly usable platform for targeted and DIA proteomics data analysis, will allow researchers to better understand the composition of complex mixtures. This will improve our ability to study the complex phenotypes associated with neurodegeneration, cancer and genetic disease.

## Supplementary Material

1

## Figures and Tables

**Table 1.** Description of datasets used in this study. mzML files for all three were accessed via Panorama [21].

Dataset	Search Engine	Peptides	Runs	Missing Fraction	Year	Citation
Alzheimer’s Disease	DIA-NN	93,610	67	0.39	2023	[14]
MMCC	DIA-NN	31,617	42	0.57	2024	[15]
TEI-REX	DIA-NN	29,503	96	0.12	2025	unpublished
