# Supplementary material for: Improved quantitative accuracy in data-independent acquisition proteomics via retention time boundary imputation
Source: bioRxiv. 2025 May 31:2025.05.27.656394. Preprint. [Version 1] doi: 10.1101/2025.05.27.656394 (PMC12154835; doi:10.1101/2025.05.27.656394)
Supplement: 1 [file NIHPP2025.05.27.656394V1-supplement-1.pdf]

bioRxiv preprint doi: https://doi.org/10.1101/2025.05.27.656394; this version posted May 31, 2025. The copyright holder for this preprint (which was not certified by peer review) is the author/funder, who has granted bioRxiv a license to display the preprint in perpetuity. It is made available under aCC-BY 4.0 International license.

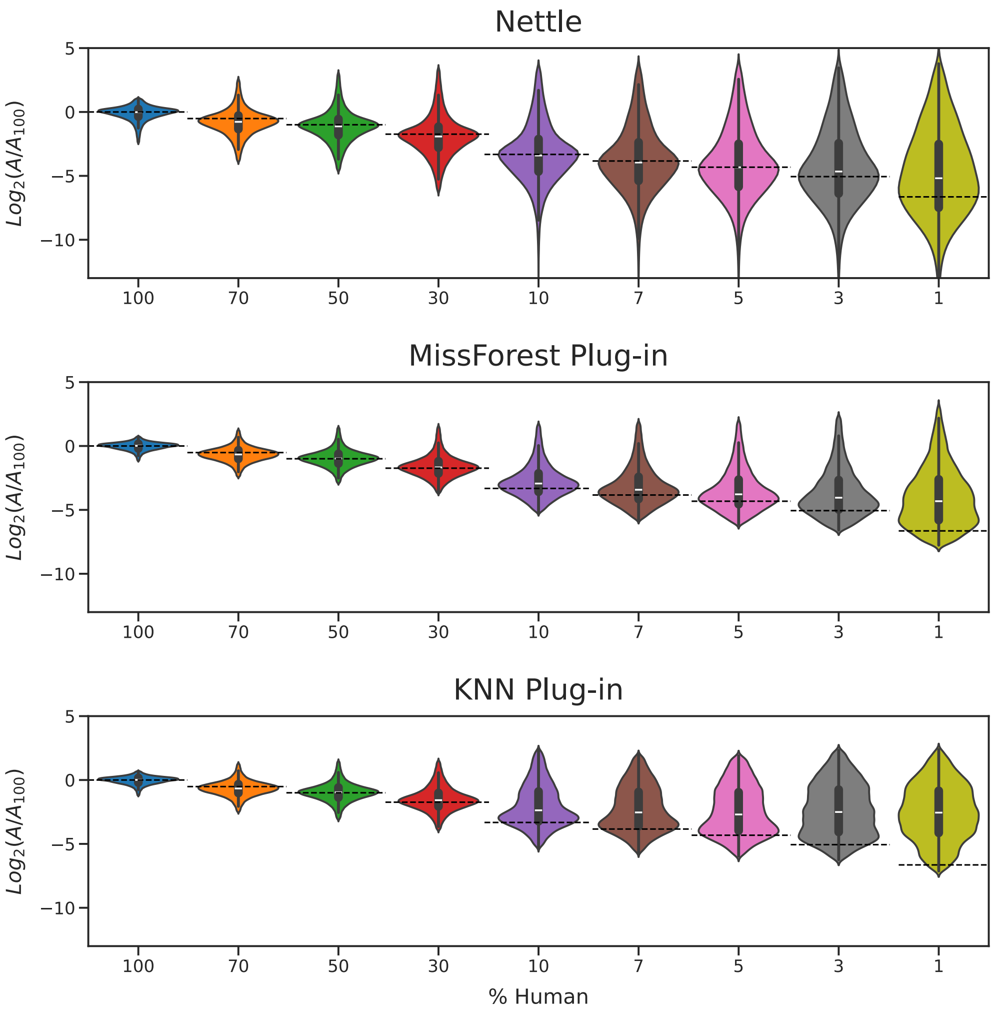

Supplementary Figure 1. **The distributions of peptide quantities after handling missing values with RT boundary or plug-in imputation.** For the MMCC experiment. Peptide abundances were normalized to the abundance in the 100% (undiluted) samples (i.e., A_{100}); $log2$ ratios are reported. The horizontal dashed lines indicate the expected ratios for each sample. The top and bottom 2% of extreme values have been removed.

bioRxiv preprint doi: https://doi.org/10.1101/2025.05.27.656394; this version posted May 31, 2025. The copyright holder for this preprint (which was not certified by peer review) is the author/funder, who has granted bioRxiv a license to display the preprint in perpetuity. It is made available under aCC-BY 4.0 International license.

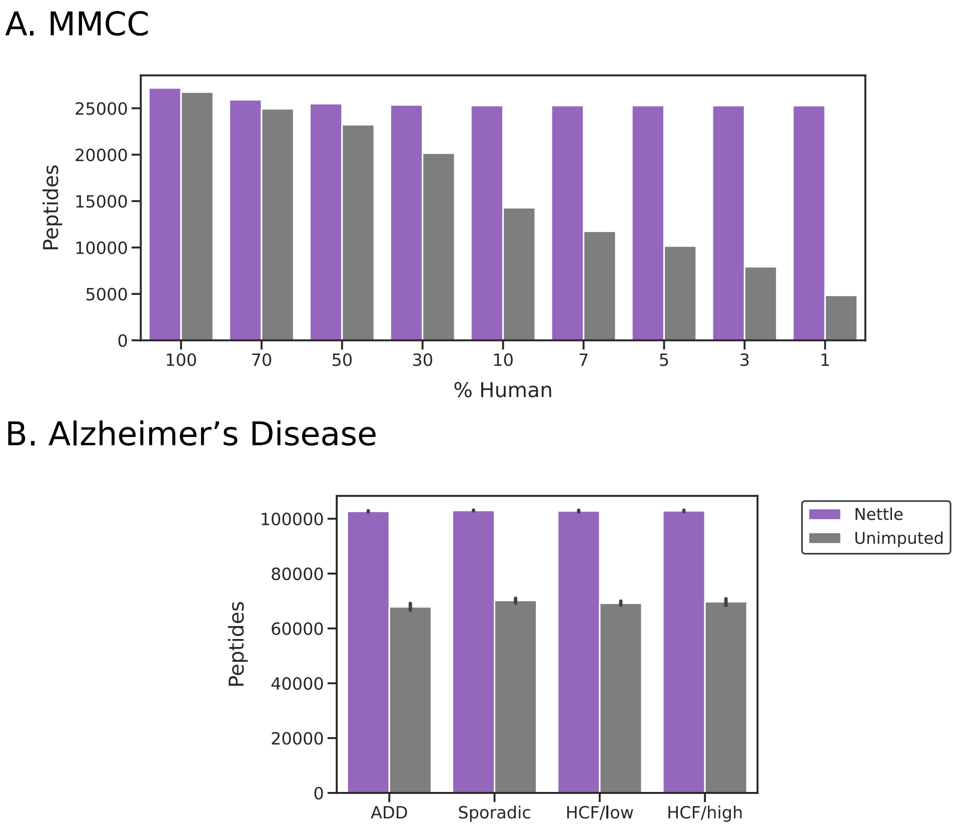

Supplementary Figure 2. **RT boundary impute increases the number of quantified peptides. A)** For the MMCC experiment. The averages of three technical replicates are shown for each dilution. **B)** For the Alzheimer's disease dataset. Quantified peptides refer to non-missing (i.e., non-NaN) peptides in each MS run.

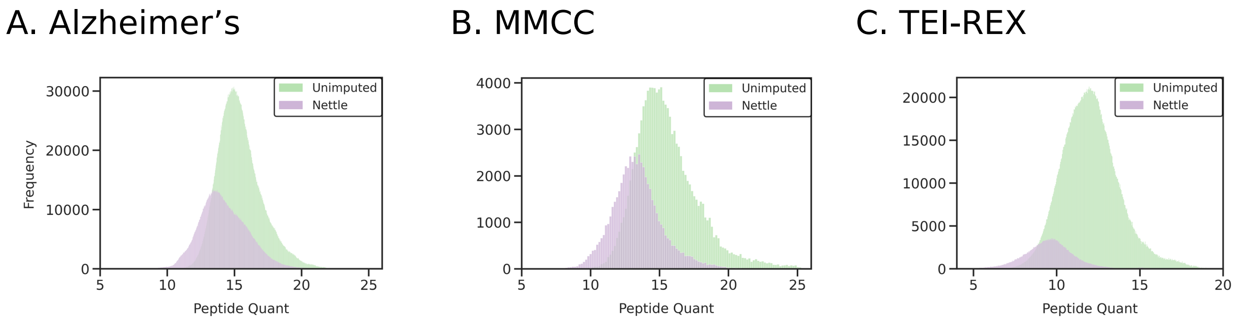

Supplementary Figure 3. **Distributions of peptide quantities before and after RT boundary imputation with Nettle. A)** For the Alzheimer's disease dataset, **B)** MMCC and **C)** TEI-REX. Peptide intensities have been $log2$ transformed.
